# Presumed cholesterinic granulomas detected on CT in horses are associated with increased lateral ventricle height and age

**DOI:** 10.1111/vru.12847

**Published:** 2020-02-05

**Authors:** Ralph A Lloyd‐Edwards, Dorien S Willems, Martijn Beukers, Astrid van den Brom‐Spierenburg, Johannes C M Vernooij, Stefanie Veraa

**Affiliations:** ^1^ Division of Diagnostic Imaging Faculty of Veterinary Medicine Utrecht The Netherlands; ^2^ Department of Equine Health Faculty of Veterinary Medicine Utrecht The Netherlands; ^3^ Department of Farm Animal Health, Faculty of Veterinary Medicine Utrecht University Utrecht The Netherlands

**Keywords:** cholesteatomas, equine, hydrocephalus, mineralisation, neurological

## Abstract

Cholesterinic granulomas are mass‐like lesions that form at the choroid plexus of the ventricular system. Large cholesterinic granulomas within the lateral ventricles have been reported to cause severe neurological signs. However, little data are available about their prevalence or appearance in the overall population. The objective was to report the prevalence of presumed cholesterinic granulomas on CT in a population of horses, and investigate associations between presumed cholesterinic granuloma presence, lateral ventricle size, age, and neurological signs. The study was cross sectional, CT scans of the head were assessed for presumed cholesterinic granuloma presence and size, and lateral ventricle height. Computed tomography findings and clinical information were compared using nonparametric testing. Computed tomography scans of 139 horses were included. Presumed cholesterinic granulomas were found in 22 horses (15.8%), nine were unilateral and 13 bilateral. A significant increase in prevalence was observed with age (*P *< .0001), with 38% of horses over 15 years old affected. The median volume of presumed cholesterinic granulomas was 242 mm^3^ with a range from 51 to 2420 mm^3^. The mean lateral ventricle height was significantly increased in horses with presumed cholesterinic granulomas present (*P* = .004), with a median of 7.3 mm compared to 4.9 mm without. Neurological signs were not associated with presumed cholesterinic granuloma presence or lateral ventricle height. Fourth ventricle mineralizations were found in seven horses, which may represent cholesterinic granulomas. In conclusion, presumed cholesterinic granulomas occurred in a large proportion of the examined population and are associated with increased lateral ventricle dilation and advanced age.

## 1 INTRODUCTION

Cholesterinic granulomas are benign masses of the choroid plexuses of horses.^1^, ^2^ They are thought to develop due to inflammation of the epithelial cell lining of the choroid plexus and consequent infiltration and deposition of cholesterol.^3^, ^4^ Cholesterinic granulomas are reported to occur in 15‐20% of older horses with no apparent clinical signs, 15% of a population of horses with epilepsy, and account for 1.3% of neoplasms detected at postmortem.^4^, ^5^, ^6^, ^7^ They are reported to cause neurological signs, which are mainly localized to the cerebrum, but also include cranial nerve deficits suggesting brainstem involvement.^1^, ^3^, ^8^ The etiology for the occurrence of the clinical signs is unknown; inflammation, compression of surrounding tissues, and obstructive hydrocephalus have all been hypothesized.^3^, ^8^, ^9^, ^10^ As a result, the characteristics that influence the relevance of cholesterinic granulomas are unknown. The ventricular system of the brain involves complex flow of cerebrospinal fluid from choroid plexus through the lateral ventricles, the third and fourth ventricle to the central canal of the spinal cord and subarachnoid space.^11^ Ventricular enlargement can occur secondarily to inflammation and obstruction both of which have been suggested in cases of cholesterinic granulomas.^3^, ^9^

The CT and MRI characteristics of cholesterinic granulomas in horses are described in a number of case reports and case series,^1^, ^3^, ^8^, ^12^ predominantly in older horses. Cholesterinic granulomas are described as usually bilateral masses of the choroid plexus in the base of the lateral ventricles, which on CT are heterogeneous, irregularly mineralized, and unreliably contrast enhance.^1^, ^2^, ^9^ Cholesterinic granulomas of the choroid plexus of the fourth ventricles are reportedly common,^4^, ^6^ although no confirmed cases on CT were found.^13^ Computed tomography is a valid choice for investigating intracranial structures of the horse, which allows assessment of the ventricular system and the presence of cholesterinic granulomas, and unlike MRI, is available in the standing horse.^1^, ^3^, ^14^, ^15^, ^16^, ^17^ Based on our review of the literature, cholesterinic granulomas are only described in clinical cases,^1^, ^2^, ^9^ and descriptions of smaller or incidental cholesterinic granulomas and relevance in a larger population are lacking in the current literature.

The objective of the study is to report the appearance and prevalence of presumed cholesterinic granulomas in a population of horses and their relevance by examining potential associations with age and gender, lateral ventricle height and symmetry, and presence of neurological signs. As such we present the following alternative hypotheses^1^: Prevalence of presumed cholesterinic granulomas increases with age,^2^ mean lateral ventricle height increases with presumed cholesterinic granuloma presence, and^3^ presence of presumed cholesterinic granulomas increases prevalence of neurological signs.

## 2 MATERIALS AND METHODS

### 2.1 Case selection

The study was cross sectional and retrospective. Computed tomographic examinations of the equine head obtained from the period of February 2015 until September 2016 in the Picture Archiving and Communication System (Impax, version 6.6.1.3004, Agfa N.V., Mortsel, Belgium) of the Division of Diagnostic Imaging (Faculty of Veterinary Medicine, Utrecht University) were retrieved. Images from client‐owned horses were used with consent given and anonymity conserved, in accordance with hospital policy with reference to research, hence, approval by Institutional Animal Care and Use Committee was not required. The horses were presented for a large variety of clinical signs associated with structures of the head, such as nasal discharge, dysphagia, trauma, or neurological signs. Examinations were included when patient history was available and the examination included the entire region of the brain. Only the original examination was included in cases when a horse underwent multiple examinations. Cases were excluded if other gross abnormalities of the brain were present, such as space occupying lesions or severe trauma. Decisions for inclusion or exclusion of cases were made by a resident in the European College of Veterinary Diagnostic Imaging (R.A.L).

### 2.2 Data recording and analysis

The CT examinations were first assessed by a European College of Veterinary Diagnostic Imaging resident (R.A.E.), who was not aware of the patient clinical signs, age, or gender at the time of evaluation. Both picture archiving and communication system and the hospital information system (Vetware, version 1.6.131‐rc01; Agfa healthcare, Rijswijk, the Netherlands) were assessed for signalment (age, breed, and gender) and history. The presence or absence of neurological signs was recorded. Neurological signs included any indication of cerebral, cerebellar, or brainstem dysfunction or impairment of cranial nerve function without evidence of this being caused by peripheral damage. Headshaking was considered a neurological sign, given the suspected involvement of the trigeminal nerve in this disease entity.^18^ Horses with solely signs of spinal cord dysfunction were not considered to have neurological signs with respect to this study. The criteria were based on the reports of cholesterinic granulomas in the veterinary literature, and those of xanthogranulomas and increased intracranial pressure in human literature.^1^, ^2^, ^9^, ^19^ The European College of Veterinary Diagnostic Imaging resident undertook initial data collection and a European College of Veterinary Internal Medicine diplomate (A.B.S.) assessed relevance of recorded neurological signs.

The images were analyzed using the viewer of the aforementioned picture archiving and communication system. The entire brain and cranial cervical spine were examined in transverse sections using a window width of 150 and window level of 40. The CT scans of the eligible cases were analyzed for any regions of increased (mineral) attenuation and more specifically the presence or absence of findings consistent with cholesterinic granulomas. Histopathology was not available for the vast majority of horses so rather than referring to cholesterinic granulomas within the lateral ventricles, we have chosen to refer to presumed cholesterinic granulomas to reflect this uncertainty.

**Figure 1:**
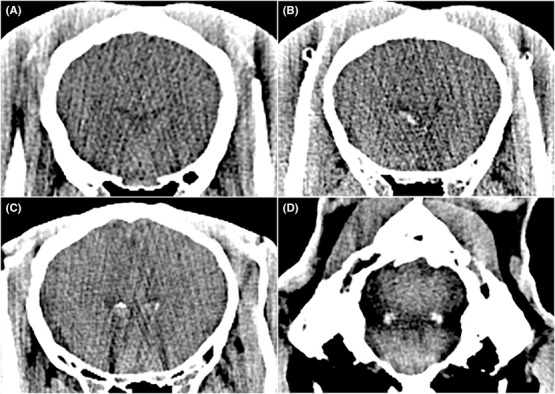
Transverse CT images at a modified soft tissue window W 150 L 40. (A), (B), and (C) are positioned just rostral to the interventricular foramen. Horse (A) has no cholesterinic granuloma present. Horse (B) has a unilateral right presumed cholesterinic granuloma and horse (C) has bilateral presumed cholesterinic granulomas present. Image (D) is positioned at the level of the lateral recesses of the fourth ventricle and is of a horse with bilateral fourth ventricle mineralizations in this region

Presumed cholesterinic granulomas were defined with reference to the current literature as partially or completely mineral attenuation, unilateral or bilateral, spheroid mass‐like lesions positioned in the axial base of the lateral ventricles.^1^, ^2^, ^9^ If a presumed cholesterinic granuloma was suspected by the initial reviewer, then the diagnosis was confirmed by a consensus of two other observers (S.V. and M.B.), both European College of Veterinary Diagnostic Imaging diplomates that were unaware of the presumed presence or absence of cholesterinic granulomas, this was achieved by blinded review. If a presumed cholesterinic granuloma was confirmed then position (left/right/bilateral) and size (length/width/height) were recorded (Figure 1). The length, width, and height of the presumed cholesterinic granulomas were measured in multiplanar reconstructions. All measurements were obtained in two planes and the mean used. The volume of the presumed cholesterinic granulomas were calculated using the formula for an ellipsoid [Volume = $\frac{4}{3}\pi \;$× (width/2) × (height/2) × (length/2)].

In absence of confirmed CT characteristics of fourth ventricle cholesterinic granulomas, any unilateral or bilateral mineralizations in the region of the lateral recesses of the fourth ventricle, with or without a mass lesion, were termed fourth ventricle mineralizations. Fourth ventricular mineralizations were recorded as present or absent. Any intracranial mineralizations that did not fit the criteria for a presumed cholesterinic granuloma or fourth ventricular mineralization were recorded and described separately. Gross pathology or histopathology findings were recorded for individuals with presumed cholesterinic granulomas or fourth ventricular mineralizations present when available.

**Figure 2:**
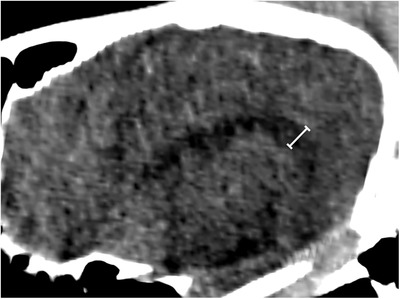
Parasagittal CT image at a modified soft tissue window width 150 and window length 40. Measurement was made as demonstrated by the line at the maximum distance from the base to floor (hippocampus or caudate nuclei) to top (corpus callosum or corona radiata) of the lateral ventricles

The lateral ventricle height was measured in a multiplanar reconstruction of a parasagittal plane. The height was defined as the greatest perpendicular distance between the ventral (hippocampus/caudate nuclei) and dorsal (corpus callosum/corona radiata) border of each lateral ventricle caudal to the interventricular foramen (Figure 2). The lateral ventricle height was recorded for each side individually, as a mean of the two sides (mean lateral ventricle height), and as a ratio of left:right lateral ventricle height. The mean lateral ventricle height was used to represent overall size of the lateral ventricles, mean lateral ventricle height to brain height ratio was used to give a measure relative to horse size, and the ratio of left to right ventricle height to allow statistical analysis of symmetry. Brain height was measured on midline from the rostrodorsal aspect of the basisphenoid bone (immediately caudal to the sphenopalatine sinus) to the dorsal aspect of the cranium. Lateral ventricle to brain height ratio was calculated by dividing the former by the latter. In addition to the first reviewer, a second blinded European College of Veterinary Diagnostic Imaging resident (D.S.W.) repeated measurements on a sample of 40 randomly chosen horses to enable reliability testing of the lateral ventricle height and presumed cholesterinic granuloma measurements.

### 2.3 Statistics

Statistical analysis was performed using a commercially available software (SPSS IBM SPSS Statistics for Windows, Version 24. IBM Corp, Armonk, NY, USA) using nonparametric tests, due to nonnormal distribution of raw and logarithmic data on a Shapiro‐Wilk test and unsatisfactory graphical assessment of a general linear model. For all testing, a *P*‐value lower than .05 was considered significant. Statistical testing was completed by an European College of Veterinary Diagnostic Imaging resident (R.A.E.), with consultation from a statistician (J.C.M.V.).

Interobserver reliability was analyzed with respect to the size of presumed cholesterinic granulomas and the measured height of both the left and right lateral ventricles using a two way mixed average measures intraclass correlation coefficient, with acceptable results being ranked from fair (0.4‐0.59), good (0.6‐0.74) to excellent (0.75‐1.0).

The presence of presumed cholesterinic granulomas (unilateral or bilateral cases were combined unless stated otherwise) was tested for association with gender using a two‐sided Fisher's exact test. Age of groups with and without presumed cholesterinic granulomas present was compared with an Independent‐Samples‐Mann‐Whitney *U* test, and a Chi‐squared test was used to test distribution within different age categories. The relationship between the volume of the presumed cholesterinic granuloma present (or largest volume if bilateral) and the age was assessed with a related‐samples Spearman's rank correlation test; for this and all following uses, correlation coefficients were ranked from “very weak” (<0.19) to “very strong” (0.8‐1.0) and an independent samples Kruskal‐Wallis test was applied to age groups.

A related‐samples Wilcoxon signed rank test significance was used to test for difference between the left and right lateral ventricle height in all horses without presumed cholesterinic granulomas present. Mean lateral ventricle height and a ratio of mean lateral ventricle height to brain height ratio were tested for association with sex using an Independent‐Samples‐Mann‐Whitney‐U test and age with a related‐samples Spearman's rank correlation test. Independent‐Samples‐Mann‐Whitney‐*U* tests were used to compare the mean lateral ventricle height and a ratio of mean lateral ventricle height to brain height ratio between cases with a bilateral presumed cholesterinic granulomas present, unilateral presumed cholesterinic granuloma present, or no presumed cholesterinic granuloma present. Asymmetrical dilation of the lateral ventricle with presumed cholesterinic granuloma presence was tested by comparing a ratio of the left:right lateral ventricle height with the presence of left and then right unilateral presumed cholesterinic granulomas, compared to presumed cholesterinic granuloma absent. Spearman's rank correlation tests were then used to examine correlation between mean lateral ventricle height and a ratio of mean lateral ventricle height to brain height ratio, to the volume of presumed cholesterinic granuloma (both total volume and volume of the largest presumed cholesterinic granuloma present were tested).

A two‐sided Fisher's exact test was used to test for association between the presence of presumed cholesterinic granulomas and neurological signs. The mean lateral ventricle height, a ratio of mean lateral ventricle height to brain height ratio, and left:right lateral ventricle height ratio was also tested for associated with to neurological signs using Independent‐Samples‐Mann‐Whitney *U* tests.

Fourth ventricular mineralizations were tested for association with age and mean lateral ventricle height (using Independent Samples‐Mann‐Whitney‐*U* tests) and a ratio of mean lateral ventricle height to brain height ratio, presumed cholesterinic granuloma presence, and neurological signs (using two‐sided Fishers exact tests).

## 3 RESULTS

### 3.1 Horses

A total of 139 CT horses were included of which 76 were mares, 59 geldings, and four stallions. The breeds included: 74 Dutch Warmbloods, 25 mixed breeds, eight Friesians, six ponies, four Haflingers, three Hannoverians, three Quarter horses, and 16 individuals of a diverse range of other breeds. The median age of the sample was 10 years (range 1‐33).

### 3.2 Imaging technical parameters

The CT scans were acquired in standing horses with a 64‐slice sliding gantry CT scanner (SOMATOM Definition AS. Siemens AG, München, Germany). The protocol utilized was 140 KVp, 328 mAs, pitch 0.8, rotation time 0.5 seconds, and 0.6 mm slice thickness. Bone (H60f) and soft tissue (H31f) reconstruction algorithms with a slice thickness of 1 and 2 mm, respectively, were applied and a standard matrix of 512 × 512 pixels used. Contrast was not utilized.

### 3.3 Interobserver reliability

Interobserver reliability was analyzed for the measured presumed cholesterinic granuloma width, height, and length finding intraclass correlation coefficents to be 0.93, 0.90, and 0.76 for the left, and 0.86, 0.63, and 0.42 for the right, respectively. All of which are excellent with the exception of height and length on the right that were good and fair, respectively. The median difference in volume on the left was 75 mm^2^ and the right 63 mm^2^. Interobserver reliability was also analyzed for measured lateral ventricle height, finding excellent agreement between observers with an intraclass correlation coefficient of 0.89 for the right and 0.91 for the left lateral ventricle height. The median interobserver difference in measured lateral ventricle height was 1.0 mm (including both left and right ventricular measurements).

### 3.4 Presumed cholesterinic granulomas

#### 3.4.1 Prevalence and size of presumed cholesterinic granulomas

Presumed cholesterinic granulomas were present in 22 horses of the 139 (15.8%, 95% confidence interval 10.5‐22.6). They were initially suspected in 24 horses, however, two cases were rejected following a consensus of expert observers, as they were not considered to completely fulfill the reported imaging criteria on the basis of a lack of clearly defined mineralization. Of the 22 horses with presumed cholesterinic granulomas, 13 were bilateral (59.1%, 95% confidence interval 38.5‐77.5) and nine unilateral (40.9%, 95% confidence interval 22.5‐61.5]). Of the unilateral presumed cholesterinic granulomas, eight were present on the left and one on the right. The median width of the measured presumed cholesterinic granulomas was 7.2 mm, height 6.9 mm, length 8.8 mm, and volume 242 mm^3^. Further measurement details are included in Table 1; volume is used for further comparisons. A wide range of volume of cholesterinic granulomas was seen with maximal volume (2420 mm^3^) measuring approximately 10 times the median and almost 50 times the minimum (51 mm^3^). A postmortem histopathological diagnosis of cholesterinic granuloma was available for one horse with bilateral presumed cholesterinic granulomas, the remaining 21 horses had no information available, all 22 cases were grouped hereafter.

**Table 1:** Median, minimum, and maximum presumed cholesterinic granulomas dimensions and volume for all cases and subdivided by those present on the right and left

	Measurement	Median	Minimum	Maximum
**Right presumed cholesterinic granulomas (n = 15)**	Width (mm)	7.2	4.4	14.1
	Height (mm)	6.9	3.7	16.7
	Length (mm)	9.4	5.5	19.6
	Volume (mm^3^)	216.0	51.0	2420.0
**Left presumed cholesterinic granulomas (n = 22)**	Width (mm)	7.5	4.8	15.9
	Height (mm)	6.9	3.8	15.7
	Length (mm)	8.8	5.2	18.3
	Volume (mm^3^)	264.0	57.7	2390.0
**All presumed cholesterinic granulomas (n = 39)**	Width (mm)	7.2	4.4	15.9
	Height (mm)	6.9	3.7	16.7
	Length (mm)	8.8	5.2	19.6
	Volume (mm^3^)	242.0	51.0	2420.0

#### 3.4.2 Association with sex and age

**Figure 3:**
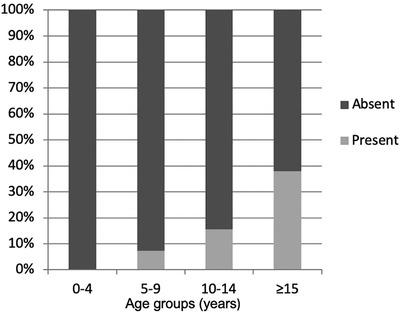
Percentage of individuals with unilateral or bilateral presumed cholesterinic granulomas in different age groups: 0‐4 years old (n = 22), 5‐9 years old (n = 41), 10‐14 years old (n = 45), and ≥15 years old (n = 29)

Presumed cholesterinic granulomas occurred in 10 geldings (16.9%, 95% confidence interval 9.1‐28.0), 12 mares (15.8%, 95% confidence interval 8.9‐25.2), and 0 stallions (0%, 95% confidence interval 0‐81), the differences in prevalence between the different gender groups were not statistically significant (*P* = .7). The median age of the horses with presumed cholesterinic granulomas present was 15 years old (range 7‐33) and absent was 9 years old (range 1‐22), two horses were of unconfirmed age so were excluded from any age‐related calculations. This age difference was statistically significant (*P* < .001). Presumed cholesterinic granulomas were present in no horses less than 5 years old (n = 22, 0%, 95% confidence interval 0‐22.8), three horses between 5 and 9 years old (n = 41, 7.3%, 95% confidence interval 2.1‐18.3), seven horses between 10 and 14 years old (n = 45, 15.6%, 95% confidence interval 7.2‐28.1), and 11 horses 15 years old and over (n = 29, 37.9%, 95% confidence interval 22.1‐56.0) (Figure 3). The different distribution of presumed cholesterinic granulomas in the age groups was statistically significant (*P* = .001).

#### 3.4.3 Correlation of volume and age

**Figure 4:**
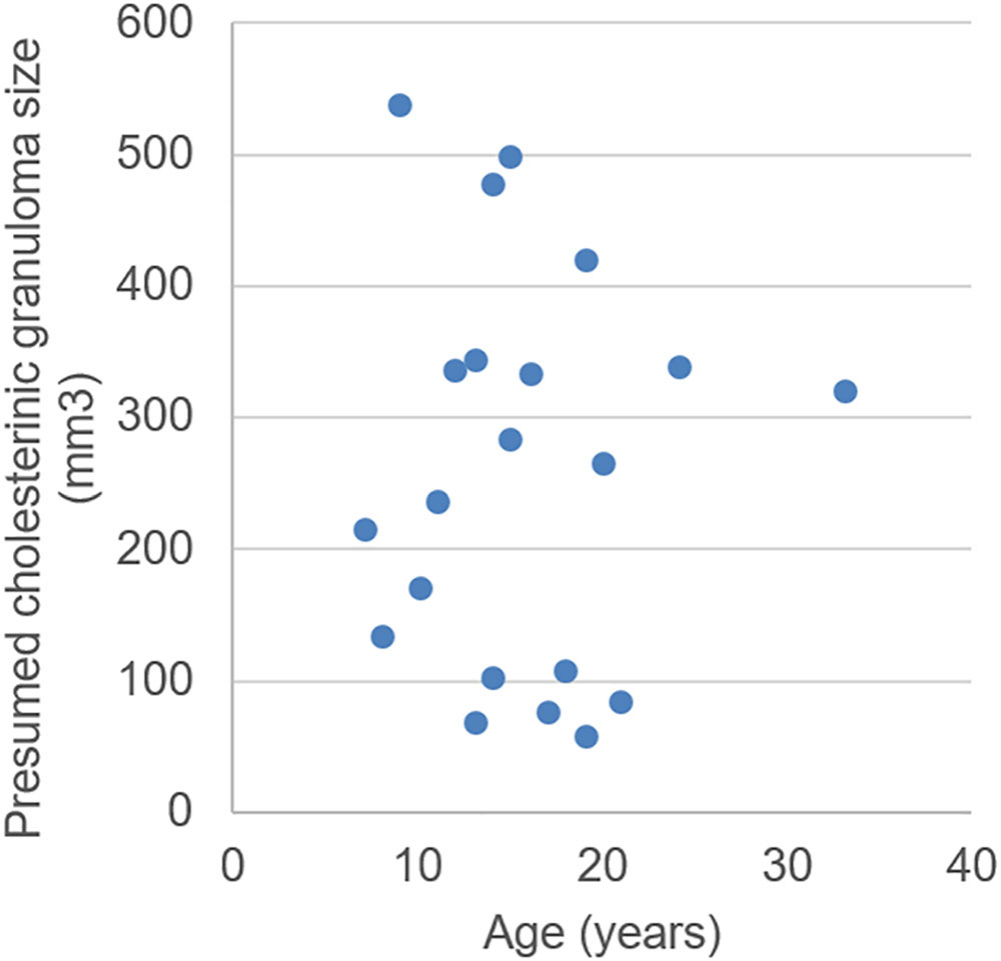
Presumed cholesterinic granuloma volume (volume of the largest if bilateral) compared to age (n = 21) [Color figure can be viewed at wileyonlinelibrary.com]

No significant associations between presumed cholesterinic granuloma volume and age groups could be found for horses with presumed cholesterinic granulomas present. A correlation coefficient of −0.065 and P = 0.8 when correlating the largest (if bilateral) presumed cholesterinic granuloma present. Largest presumed cholesterinic granuloma volume was further assessed within ordinal age groups with no significant differences found (P = 0.9)(Figure 4).

### 3.5 Lateral ventricle height

#### 3.5.1 Lateral ventricle height in horses without cholesterinic granulomas

The median “mean lateral ventricle height” was 4.9 mm and “mean lateral ventricle height to brain height ratio” was 0.053 in horses without presumed cholesterinic granulomas (Table 2). In horses without presumed cholesterinic granulomas present, the median left lateral ventricle height (5.0 mm) was significantly greater than the right (4.6 mm; *P* = .02). The median “mean lateral ventricle height to brain height ratio” in horses without presumed cholesterinic granulomas present was 0.044 in horses 0‐4 years old (n = 20), 0.059 in 5‐9 years old (n = 38), 0.056 in 10‐15 years old (n = 38), and 0.053 in horses over 15 years old (n = 18). In horses without presumed cholesterinic granulomas present the “mean lateral ventricle height” showed no correlation with age (correlation coefficient = 0.1 and *P* = .3). The mean lateral ventricle height to brain height ratio did show significant correlation with age (correlation coefficient 0.19 and *P* = .05). The correlation disappeared after exclusion of horses of 0‐4 years old (correlation coefficient 0.073 and *P* = .4), The exclusion was performed to enable assessment of only age groups with presumed cholesterinic granulomas and hence remove any correlation with age as a possible source of interference in further comparisons.

**Table 2:** Median, maximum, and minimum values of the mean lateral ventricle height and left:right ratio; divided into groups with absent presumed cholesterinic granuloma, bilateral presumed cholesterinic granulomas or unilateral presumed cholesterinic granulomas (left and right unilateral results are combined for mean lateral ventricle as it is a combined measurement of both sides)

Measurement	Presumed cholesterinic granuloma	n	Median (mm)	Minimum (mm)	Maximum (mm)
Mean lateral ventricle height	Absent	117	4.9	1.4	15.9
	Unilateral	9	6.7	3.7	11.2
	Bilateral	13	7.5	3.2	13.0
Ratio left:right lateral ventricle height	Absent	117	1.0	0.4	10.8
	Unilateral right	1	0.7	0.7	0.7
	Unilateral left	8	0.9	0.6	2.3
	Bilateral	13	1.00	0.4	2.0

#### 3.5.2 Association of lateral ventricle height with presumed cholesterinic granulomas

**Figure 5:**
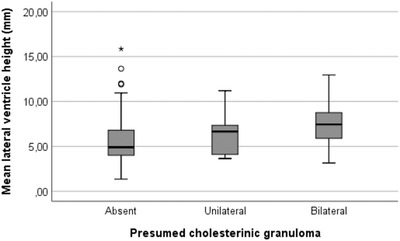
Mean lateral ventricle height in cases with absent (n = 117), unilateral (n = 9), or bilateral (n = 13) presumed cholesterinic granulomas present

The median “mean lateral ventricle height” was 7.3 mm and median “mean lateral ventricle height to brain height ratio” was 0.077 in horses with presumed cholesterinic granulomas, both of which were significantly greater than those with no presumed cholesterinic granulomas (*P* = .004 and *P* = .001, respectively). The significant difference was maintained after exclusion of all horses 0‐4 years old (*P* = .01 and *P* = .006, respectively). No significant difference between horses with unilateral or bilateral presumed cholesterinic granulomas was found (*P* = .5 and *P* = .4 for the absolute and ratio values, respectively; Figure 5). No significant correlation between the volume of the presumed cholesterinic granuloma and mean lateral ventricle height was found (for total presumed cholesterinic granuloma volume correlation coefficient = 0.17 and *P* = .7, and largest presumed cholesterinic granuloma present correlation coefficient = 0.15 and *P* = .5).Similarly no significant correlations were found with mean lateral ventricle height to brain height ratio with largest (correlation coefficient 0.18 and *P* = .4) or total (correlation coefficient 0.19 and *P* = .4) presumed cholesterinc granuloma volume. The ratio of left:right lateral ventricle height was insignificantly altered in horses with unilateral left (*P* = .6), unilateral right (*P* = .1) or bilateral presumed cholesterinic granulomas (*P* = .5) present compared to horses without presumed cholesterinic granulomas (Table 2).

#### 3.5.3 Neurological signs

Neurological signs were seen in 25 horses of the total of 139, of which nine displayed headshaking, six vestibular/cerebellar ataxia, five cranial nerve deficits, four abnormal mentation, and two epilepsy. Neurological signs showed no significant association with sex or age (*P* = .6 and *P* = .3, respectively). Neurological signs were seen in two of the 22 cases with presumed cholesterinic granulomas present (9.1%, 95% confidence interval 1.9‐26.1) and 23 of the 115 cases without presumed cholesterinic granulomas (19.7%, 95% confidence interval 13.2‐27.5); the difference in occurrence was not significant (*P* = .4). The two horses with neurological signs and presumed cholesterinic granulomas presented differently, one presented with dysphagia (dental origin was suspected) and suspected cranial nerve deficits and the other headshaking. Both had bilateral presumed cholesterinic granulomas: the right presumed cholesterinic granulomas measured 537 and 133 mm^3^, respectively, the left presumed cholesterinic granulomas of 483 and 122 mm^3^, respectively, and the latter also had bilateral fourth ventricular mineralizations. No significant associations of neurological signs with mean lateral ventricle height, mean lateral ventricle height to brain height ratio, or the ratio of left:right lateral ventricle height were found (*P* = .4, *P* = .9, and *P* = .5, respectively)

### 3.6 Fourth ventricle and other intracranial mineralizations

Fourth ventricle mineralizations were found bilaterally in seven horses (5%, 95% confidence interval 2.3‐9.6), no unilateral cases were observed. All incidences were seen as mineralizations, without a defined mass lesion presumably due to border effacement with the surrounding brain parenchyma (in contrast to presumed cholesterinic granulomas in the lateral ventricles that were predominantly surrounded by cerebrospinal fluid). The median age of horses with fourth ventricular mineralizations present (17 years, range 9‐33) was significantly greater than those without (10 years, range 1‐24; *P* = .02).

#### 3.6.1 Association of fourth ventricle mineralizations with presumed cholesterinic granulomas

No association between fourth ventricle mineralization presence and neurological signs was found (*P* = 1.0). Fourth ventricle mineralizations were significantly more likely to occur in horses with presumed cholesterinic granulomas present (*P* = .001); five of the seven horses with fourth ventricular mineralizations also had presumed cholesterinic granulomas present (two unilateral and three bilateral). The median “mean lateral ventricle height” was 7.15 mm in horses with fourth ventricle mineralizations compared to 5.13 mm without, the difference did not reach a level of significance (*P* = .1). Similarly, the difference between the median lateral ventricle height to brain height ratios (0.055 in horses without and 0.78 for horses with fourth ventricular mineralizations) did not reach significance (*P* = .7).

### 3.7 Other mineralizations

Additionally, several intracranial mineralizations were found that did not fit the criteria for presumed cholesterinic granulomas or fourth ventricular mineralisations. These included: bilateral mineralizations within the ventral piriform lobes of three horses, a small mineralization in the white matter of the frontal lobe in a single horse, and a central 1 mm diameter mineralization of the brain stem in one horse.

## 4 DISCUSSION

This study has further investigated the presence and possible relevance of presumed cholesterinic granulomas as a common incidental finding on CT examination of the head. Among other observations, our findings lead to the acceptance of the first hypothesis that prevalence of presumed cholesterinic granulomas increases with age, acceptance of the second hypothesis that mean lateral ventricle size increases with presumed cholesterinic granuloma presence, and rejection of the final hypothesis that prevalence of neurological signs increases with presumed cholesterinic granuloma presence.

The CT presentation of presumed cholesterinic granulomas in the present study concur with the appearance of cholesterinic granulomas in present literature: predominantly bilateral (59%) oval masses in the base of the rostral to mid aspect of the lateral ventricles, with irregular hyperattenuating (mineral) aspects in their periphery.^1^, ^8^ However, all previously reported cases of cholesterinic granulomas tend to describe larger masses. No presumed cholesterinic granulomas detected in this study reached the sizes described. The median width of detected presumed cholesterinic granulomas in this study was found to be of 7.2 mm and maximum width of 15.9 mm. Whereas in all previously reported cases, they were much larger with a minimum width of ± 3 cm, inferring that the vast majority of presumed cholesterinic granulomas present in the population are likely to be much smaller than those previously reported. The authors consider this to be the most likely reason for the lack of any correlation of neurological signs and presumed cholesterinic granuloma presence and rejection of the third hypothesis, despite neurological signs reported in previous literature.^1^, ^3^, ^8^, ^9^

The prevalence of cholesterinic granulomas has previously been a subject of disagreement, with prevalence reported as 15‐20% in older horses^4^ or 15% of a population of epileptic horses.^6^ Our study found the overall prevalence of presumed cholesterinic granulomas in the sample population of 15.8% and a much higher prevalence in older horses (15 years or older) of 40%. This difference could be due to altered rates of postmortem detection rather than a representative live population^4^, ^5^ or differences in region and demographic. Breed was not examined in the current study as an uneven distribution, prevented accurate comparisons.

A significant difference in prevalence of presumed cholesterinic granulomas between younger and older horses was confirmed leading to acceptance of our first hypothesis. No significant association between age and the size of presumed cholesterinic granuloma was found, which would seem to concur with a previous paper that examined a case of bilateral cholesterinic granulomas over time and found an overall reduction in size.^2^ Although further longitudinal CT data collection is required to make meaningful conclusions about change in cholesterinic granuloma size over time.

Cholesterinic granulomas have been suggested to cause neurological signs due to primary pressure changes, and consequent to lateral ventricle dilation and hydrocephalus.^9^ For this reason, lateral ventricle height was also investigated. This is the first study to describe lateral ventricle height in horses. Lateral ventricle height is a simple and repeatable radiological measure of lateral ventricle size, it avoids the confounding presence of presumed cholesterinic granuloma within the measurement region and has been demonstrated as effective as area and volume in detecting hydrocephalus in dogs.^20^ The method of measurement of lateral ventricle height and brain height used were subtly altered from the method used in dogs, as MRI enabled accurate assessment of both values in a single plane, however, relatively limited soft tissue detail necessitated choosing the greatest height of the lateral ventricle for measurement rather than a simple midline height. Additionally, bony rather than soft tissue landmarks were chosen for brain height to increase reliability of measurement on CT.^20^, ^21^ The repeatability of the measure was confirmed by excellent interobserver reliability testing. The lateral ventricle median height was 4.9 mm, and significantly greater on the left than right side. This finding coincides with previous studies in dogs finding that the left lateral ventricle is larger than the right in the majority of cases.^22^ Overall results between absolute lateral ventricle height and a ratio of lateral ventricle height to brain height varied little. However, the ratio did reveal a significant increase in relative lateral ventricle height with increased age. This could be due to reduction in brain volume with age a phenomenon previously reported in dogs.^21^

Mean lateral ventricle height was significantly increased in cases with either a bilateral or unilateral presumed cholesterinic granulomas. This would appear to support the suggestion that lateral ventricle dilation occurs secondarily to cholesterinic granuloma presence.^3^, ^9^ However, within the population of horses with a presumed cholesterinic granuloma present a significant positive correlation between presumed cholesterinic granuloma size and mean lateral ventricle size was not present, and the ratio of left:right lateral ventricle height was not altered in cases with unilateral presumed cholesterinic granulomas. This suggests the absence of a straightforward causal relationship between cholesterinic granuloma presence and lateral ventricle obstruction, and therefore increased size as previously suggested^9^; instead it may be due to differences in inflammatory status of the choroid plexus and therefore cerebrospinal fluid production or resorption.^2^

Histopathology confirmed presumed cholesterinic granulomas in one horse. Although histopathology was not available in the remaining horses the authors believe their choice of referring to the described processes as presumed cholesterinic granulomas is accurate and representative, due to the close adherence in imaging characteristics to the previously described cases, and relative lack of other reported differentials. In addition to cholesterinic granulomas; choroid plexus carcinoma, choroid plexus papilloma, and papillary ependymomas can also cause space occupying lesions of the choroid plexus.^12^, ^23^, ^24^, ^25^, ^26^, ^27^ Based on our review of the literature, there are no reported cases of choroid plexus carcinoma in the horse. Rare cases of choroid plexus papilloma and papillary ependymoma are reported in the horse,^12^, ^23^, ^24^, ^27^ however, in neither process in horses has mineralization been described as a feature, which was a key part of our utilized criteria. Additionally, 59% of observed presumed cholesterinic granulomas were bilateral, which is not a reported feature of any of the differentials. Hence, it seems reasonable that by examining CTs for structures using our described criteria by a consensus of three qualified individuals, masses that in all likelihood are consistent with cholesterinic granulomas can be detected.

Contrary to recent literature,^1^, ^2^, ^3^, ^8^ cholesterinic granulomas have previously been quoted as being most common in the fourth rather than lateral ventricles.^4^, ^6^ Bilateral fourth ventricular mineralizations were found at the level of the choroid plexus in the lateral recesses of seven horses. These are considered to most likely represent cholesterinic granulomas due to position and bilateral presentation. However, at present, CT and pathological correlation are limited in the literature unlike those found in the lateral ventricles. A mineralized fourth ventricle cholesterinic granuloma has been described but not confirmed in a horse.^13^ One case was confirmed in a dog, however, that case presented with a large central mass like lesion.^28^ As such other differentials such as other choroid plexuses tumours (papilloma etc) or siderocalcinosis are possible.^29^ Siderocalcinosis is considered as a differential, despite its occurrence in the white matter of the cerebellum not choroid plexus. This is due to the limited soft tissue contrast on CT images, due to the minimal amount of observed fluid in the lateral recesses of the fourth ventricles and the location adjacent to the petrous temporal bone.^14^, ^29^ The piriform lobe mineralization is of unknown relevance.

Although this study increases understanding of prevalence and characteristics of cholesterinic granulomas, it has limitations that require further investigation. The major limitation is the limited histopathological correlation of CT findings, particularly in the region of the fourth ventricular mineralizations and lack of literature on this matter. Additionally, the study is based on CT studies of individuals at a single time point (multiple examinations were available in some horses within our population but none with cholesterinic granulomas present); hence development and progression of the lesions is unknown. A wider scale study into horses exhibiting neurological signs linked to cholesterinic granulomas would be interesting to assess relevance of findings. Specifically, at what size presumed cholesterinic granulomas or cholesterinic granulomas cause neurological signs, or if the presence or absence of neurological signs are more a reflection of inflammatory status (and secondarily lateral ventricle size) of the choroid plexus. Magnetic resonance imaging may give more functional information (cerebrospinal fluid composition, edema) and therefore may allow better assessment of the clinical relevance of cholesterinic granulomas^14^ but its use is limited due the need of general anesthesia.

In conclusion, presumed cholesterinic granulomas occur in the lateral ventricle of 15.8%, of the sample population and 38% of horses over 15 years old. Presumed cholesterinic granulomas significantly increased in prevalence but not size with advanced age. The mean lateral ventricle height significantly increased in cases with presumed cholesterinic granulomas present. However, association between lateral ventricle height and presumed cholesterinic granuloma size was not present and the left: right lateral ventricle height ratio did not significantly vary in unilateral cases. No evidence of association between presumed cholesterinic granuloma presence or lateral ventricle height with neurological signs was elucidated. Fourth ventricular mineralizations were also found in 5% of horses most of which also had lateral ventricle presumed cholesterinic granulomas. Future longitudinal studies are needed to confirm the correlation between cholesterinic granuloma development, lateral ventricle size, and inflammation status of the ventricular system/choroid plexus and its implication on clinical signs.

## LIST OF AUTHOR CONTRIBUTIONS

### Category 1

(a) Conception and Design: Lloyd‐Edwards, Beukers, Veraa
(b) Acquisition of Data: Lloyd‐Edwards, Willems, Brom‐Spierenburg,
(c) Analysis and Interpretation of Data: Lloyd‐Edwards, Brom‐Spierenburg, Vernooij,

### Category 2

(a) Drafting the Article: Lloyd‐Edwards,
(b) Revising Article for Intellectual Content: Lloyd‐Edwards, Willems, Beukers, Brom‐Spierenburg, Vernooij, Veraa

### Category 3

(a) Final Approval of the Completed Article: Lloyd‐Edwards, Willems, Beukers, Brom‐Spierenburg, Vernooij, Veraa

## CONFLICT OF INTEREST DISCLOSURE

None of the authors have conflicts of interest to disclose.
